# The efficacy of pineapple juice as a negative oral contrast agent in magnetic resonance cholangiopancreatography

**DOI:** 10.4102/sajr.v24i1.1875

**Published:** 2020-07-29

**Authors:** Sheryl Mohabir, Richard D. Pitcher, Rubeshan Perumal, Matthew D.M. Goodier

**Affiliations:** 1 Division of Radiodiagnosis, Department of Medical Imaging and Clinical Oncology, Faculty of Medicine and Health Sciences, Stellenbosch University, Cape Town, South Africa; 2 Centre for the AIDS Programme of Research in South Africa, University of KwaZulu-Natal, Durban, South Africa; 3 Department of Radiology, Grey’s Hospital Pietermaritzburg, University of KwaZulu-Natal, KwaZulu-Natal, South Africa

**Keywords:** MRCP, pineapple juice, negative oral contrast, pancreatobiliary ducts, common bile duct, pancreatic duct

## Abstract

**Background:**

During magnetic resonance cholangiopancreatography (MRCP), the pancreatobiliary ducts can be obscured by the high-intensity signal from the stomach and duodenum. Pineapple juice may be an alternative to commercially available negative contrast agents, but has not been evaluated locally.

**Objectives:**

To evaluate the efficacy of a local, off-the-shelf pineapple juice preparation as a negative oral contrast agent for MRCP.

**Method:**

An observational, analytical study was conducted during January–December 2017. A 1.5 Tesla MRCP sequence was performed immediately before and after ingestion of 250 mL of a local, commercially-available pineapple juice preparation. Image evaluation was performed by two radiologists with independent, blind assessment of gastric/duodenal signal intensity and biliary /pancreatic duct visibility, before and after pineapple juice.

**Results:**

Fifty adult patients (F = 44, 88%) with median age 44 years (IQR: 34.75, 57) were included. After pineapple juice administration, there was significant measured (1661.51 vs. 1409.94, *p* < 0.01) and perceived (2.16 vs. 2.72, *p* < 0.01) duodenal signal reduction but no significant change in measured (1081.17 vs. 1044.38, *p* = 0.34) or perceived (2.73 vs. 2.84, *p* = 0.14) gastric signal intensity. Visibility of the common bile duct was significantly improved (3.67 vs. 3.86, *p* < 0.01), whilst that of the main pancreatic duct showed no significant change (2.92 vs. 2.86, *p* = 0.44).

**Conclusion:**

The local pineapple juice preparation used in this study is an effective, affordable and natural negative oral contrast agent for enhancement of MRCP images, and specifically improves visualisation of the common bile duct.

## Introduction

Magnetic resonance cholangiopancreatography (MRCP) is a non-invasive technique for imaging the biliary and pancreatic ducts.^1^ Although endoscopic retrograde cholangiopancreatography (ERCP) has enhanced the evaluation of these ducts over the past 50 years and has both diagnostic and therapeutic capacity,^2^ it is invasive, utilises ionising radiation, has relatively high morbidity and requires special preparation, as well as sedation.^3,4^ Despite endoscopic ultrasound (EUS) achieving 88% – 97% sensitivity and 96% – 100% specificity for choledocholithiasis, its role in this domain is not well established.^5^ As MRCP has diagnostic accuracy comparable to ERCP, it is preferred for initial pancreaticobiliary investigation, with ERCP reserved for therapeutic interventions and biopsy.^6,7,8,9,10,11,12^

Magnetic resonance cholangiopancreatography utilises heavily T2-weighted, mainly coronal, maximum intensity projections (MIP), similar to conventional cholangiopancreatography.^13^ However, the high-intensity signal from adjacent gastric and duodenal fluids may obscure the bile and pancreatic ducts, limiting diagnostic accuracy.^14,15^ To overcome this, patients are fasted before the examination and a negative oral contrast agent may be administered to further suppress signal from these structures.^16^ Negative contrast agents use the paramagnetic properties of heavy metal ions, particularly manganese, barium and gadolinium, to reduce T1 and T2 relaxation times, thereby increasing T1- and decreasing T2-signal intensity.^17,18^ Commercially available agents have been shown to significantly improve visualisation of the bile and pancreatic ducts, with no impact on duct dimensions.^19^ A systematic review of oral negative contrast agents for improving visualisation of the hepatobiliary system during MRCP, which included 25 studies over 25 years from 1990 to 2015, revealed that the application of an oral substance for gastrointestinal signal suppression was effective in 23 out of the 25 studies.^20^ However, these are expensive, not widely available and generally unpalatable, restricting their use, especially in resource-limited settings.

The ideal oral contrast agent should be affordable, palatable and evenly distributed in the gastrointestinal tract; it should not be toxic, diluted during transit or stimulate peristalsis.^21^ There is an ongoing quest for a cheaper, more palatable, but effective, natural alternative to commercially available negative MRCP contrast agents.

Pineapple is the fruit with the highest manganese concentration.^22^ Five prior studies from Europe (*n* = 4) and South America (*n* = 1) with cohorts between 10 and 100 subjects have assessed commercially available pineapple juice as a negative MRCP contrast agent.^22,23,24,25,26^ Three studies used volumes more than 400 mL, whilst two studies used 180 mL, in combination with commercially available gadolinium contrast. Pineapple juice with manganese concentrations greater than 15 mg/L was found to be comparable to the commercially available negative contrast agent ferumoxsil.^23^

Pineapple juice is routinely used as a negative oral MRCP contrast agent at Grey’s Hospital, a 530-bed public-sector tertiary-level teaching hospital in Pietermaritzburg, Kwazulu-Natal, South Africa. However, the efficacy of the institutional MRCP protocol has not been formally evaluated. The aim of this study was, therefore, to assess the role of an off-the-shelf, conveniently packaged (carton with attached straw), easily consumable quantity (250 mL) of pineapple juice as a negative oral contrast agent for MRCP within a resource-constrained specialist diagnostic radiology service.

## Methods

An observational, comparative, analytical study was conducted within the radiology department at Grey’s Hospital in KwaZulu-Natal, South Africa. The study population included all adult patients (18 years and older) undergoing MRCP with pineapple juice as a negative contrast agent, from 01 January to 31 December 2017. Patients with a history of a previous gastrectomy were excluded.

**FIGURE 1:**
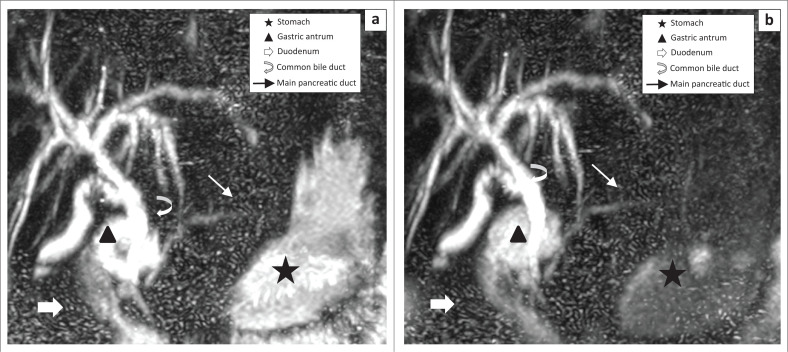
This maximum intensity projection coronal reconstruction magnetic resonance cholangiopancreatography image demonstrates a significant reduction in signal intensity of the stomach from moderate to slight signal intensity (Δ maximum ROI from 1883.5 to 1020): (a) pre-contrast magnetic resonance cholangiopancreatography; (b) post-contrast magnetic resonance cholangiopancreatography. Correspondingly, visualisation of the common bile duct (CBD) improved from good to excellent. This is explained by reduction in signal in the gastric antrum resulting in improved visualisation of the overlapping CBD. The duodenum had no subjective improvement with moderate signal intensity in both pre- and post-contrast imaging (Δ maximum ROI 2145 → 1897). The main pancreatic duct (MPD) remained visible with some difficulty.

**FIGURE 2:**
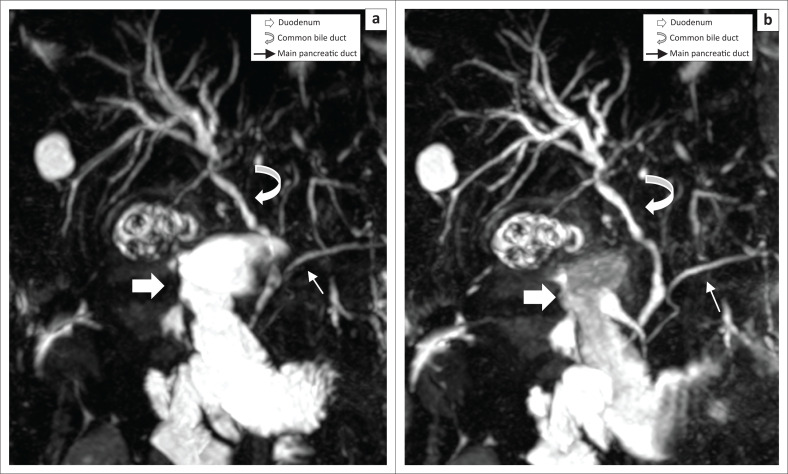
This maximum intensity projection coronal reconstruction magnetic resonance cholangiopancreatography image demonstrates a significant reduction in the signal intensity of the duodenum from high to moderate signal intensity (Δ maximum ROI 2230 → 2192): (a) pre-contrast magnetic resonance cholangiopancreatography; (b) post-contrast magnetic resonance cholangiopancreatography. Correspondingly, visualisation of the common bile duct (CBD) improved from fair to excellent, and visualisation of the main pancreatic duct (MPD) improved from good to excellent.

Information relating to patients’ age, gender and indication for MRCP were recorded. All patients fasted for a minimum of 10 h before the procedure. Standard MRCP sequences before and immediately after oral administration of 250 mL of pineapple juice (Liqui Fruit Summer Pine, Ceres, South Africa) were completed on all patients, utilising a 1.5 Tesla MR Unit (Philips Intera System, Amsterdam, Netherlands) with a SENSE body coil. The pre- and post-contrast images (examples provided in Figures 1 and 2) were re-labelled by a randomisation table to facilitate a blinded review.

Images were assessed by two radiologists: a consultant with more than five years’ experience and a senior radiology trainee. Signal intensity (SI) of the stomach and duodenum was measured on pre- and post-contrast images, using the Carestream picture archiving and communication system (PACS). A 100 mm^2^ region of interest (ROI) was used to assess maximum gastric SI on MIP, whilst a smaller, oval ROI was used for the duodenum. The overall perceived SI of the stomach and duodenum were recorded using a 4-point Likert scale (Table 1). The visibility of the common bile duct (CBD) and main pancreatic duct (MPD) before and after pineapple juice administration were graded qualitatively using the 4-point Likert method (Table 2).

**TABLE 1:** Perception of signal intensity in the stomach and duodenum.

Score	Signal suppression in the stomach and duodenum
1	Poor (high signal intensity)
2	Fair (moderate signal intensity)
3	Good (slight signal intensity)
4	Excellent (no signal intensity anywhere)

**TABLE 2:** Visibility and detectability of common bile duct or main pancreatic duct.

Score	Visibility	Detectability of CBD and MPD
1	Poor	Not detected
2	Fair	Difficult to detect the anatomy
3	Good	The anatomy is visible but with some difficulty
4	Excellent	Completely visible

CBD, common bile duct; MPD, main pancreatic duct.

All data were analysed using R 3.5.3. For all statistical comparisons, a 5% level of significance was used. All data were assessed for normality, and non-parametric tests were used where necessary. Inter-rater agreement and reliability were assessed using Kendall’s coefficient of concordance and the intraclass correlation coefficient. A mixed-model two-way analysis of variance (ANOVA) was used to compare the measured and perceived difference in SI before and after pineapple juice ingestion, and to identify the changes in the visualisation of the CBD and MPD.

### Ethical consideration

This study was approved by the Biomedical Research Ethics Committee of the University of KwaZulu-Natal (BE622/16) and the Kwazulu-Natal Provincial Health Research and Ethics Committee (KZ_2016RP43_873). Informed consent was obtained from all patients involved in this study.

## Results

A total of 50 patients were included in the study; the median age was 44 years (IQR 43.75, 57), and the majority (88%) were women. The most common indication for MRCP was suspected choledocholithiasis or benign biliary strictures (72%), whilst post-surgical evaluation (20%) was the next most common indication.

After pineapple juice administration, there was significant measured (1661.51 vs. 1409.94, *p* < 0.01) and perceived (2.16 vs. 2.72, *p* < 0.01) duodenal signal reduction but no significant change in measured (1081.17 vs. 1044.38, *p* = 0.34) or perceived (2.73 vs. 2.84, *p* = 0.14) gastric SI (Table 3). Visibility of the CBD was significantly improved (3.67 vs. 3.86, *p* < 0.01), whilst that of the MPD showed no significant change (2.92 vs. 2.86, *p* = 0.44).

**TABLE 3:** Effect of pineapple juice as a negative contrast agent in the evaluation of pancreatobiliary structures by magnetic resonance cholangiopancreatography.

Characteristics	Pre-contrast		Post-contrast		p*
	Mean	SD	Mean	SD	
SI Stomach (ROI)	1081.17	479.75	1044.38	404.33	0.34
SI Duodenum (ROI)	1661.51	826.87	1409.94	741.03	< 0.01
SI Stomach (Subjective)	2.73	0.78	2.84	0.53	0.14
SI Duodenum (Subjective)	2.16	0.91	2.72	0.81	< 0.01
MPD	2.92	0.99	2. 87	1.05	0.44
CBD	3.67	0.67	3.86	0.38	< 0.01

* , Mixed model ANOVA, two-way.

SD, standard deviation; CBD, common bile duct; MPD, main pancreatic duct; ROI, region of interest; SI, signal intensity.

There was substantial agreement between reviewers on all study items: measured SI from the stomach (*κ* = 0.97, 95% confidence interval [CI] 0.96–0.98) and the duodenum (*κ* = 0.88, 95% CI 0.83–0.92); perceived SI from the stomach (*κ* = 0.81, 95% CI 0.73–0.87) and the duodenum (*κ* = 0.9, 95% CI 0.86–0.93); subjective assessments of the visibility of the CBD (*κ* = 0.88, 95% CI 0.83–0.92) and MPD (*κ* = 0.91, 95% CI 0.86–0.94).

## Discussion

To our knowledge, this is one of the few studies to date that quantitatively evaluates changes in MRCP SI after pineapple juice administration.^22,23,24,25,26^ Our finding that 250 mL of an affordable, local, off-the-shelf pineapple juice preparation decreased duodenal SI by 15% and significantly enhanced CBD visualisation points to its efficacy as an MRCP negative oral contrast agent in our setting. This has general applicability in South Africa and more broadly in Africa, where pineapple juice is readily available and affordable. Our findings thus have the potential to influence MRCP protocols on our continent.

The only prior study that quantified SI changes following pineapple juice administration was confounded by the off-label co-administration of 1 mL of oral gadolinium, a potent synthetic negative contrast agent.^24^ This study achieved 96% and 90% quantitative loss of gastric and duodenal fluid SI, respectively, and a mean qualitative improvement of 2 points on a 4-point Likert scale, similar to that used in our study. However, the safety, efficacy and feasibility of oral gadolinium have yet to be definitively assessed.^24,27^ Although the degree of duodenal signal suppression was substantially lower than the aforementioned study, the use of pineapple juice in this study resulted in 86% (vs. 76% without pineapple juice) of our images displaying excellent visualisation of the CBD.

The volume of pineapple juice administered in this study was that of a commercially packaged juice carton with an attached straw (250 mL) which was convenient for consumption in the supine position. Most *in vivo* studies to date have administered 400 mL of pineapple juice, although the exact amount of manganese has not been disclosed.^22,23^ Electrothermal atomic absorption spectrometry assay demonstrated a manganese concentration of 27.6 mg/L for pure pineapple juice.^22^ The concentration of manganese in commercially available pineapple juice preparations ranges from 9.3 mg/L to 12.7 mg/L, which, although substantially lower than the 40 mg/L manganese concentration achieved with commercially prepared manganese contrast agents, was shown to be effective for elimination of signal from the digestive tract.^19,26^ Studies of blueberry juice as a negative MRCP contrast agent report effective manganese concentrations of 19 mg/L^28^ and 30 mg/L^29^, which is only achieved with volumes of 430 mL and 600 mL of most commercially available blueberry juices, respectively. In addition, 100 mL of date syrup can be used as an effective negative contrast agent at MRCP, with adequate suppression of signal from the gastrointestinal system, improved visualisation of the biliary system and increased calibre of the pancreatic duct because of the acidic nature of date syrup.^30^

The ideal dose of pineapple juice may similarly be dependent on the concentration of manganese and the propensity of the dose to accumulate in the stomach and duodenum whilst displacing gastrointestinal fluids. The lower volume of pineapple juice administered in our study, in comparison to the volume administered in most other studies, may account for poor signal suppression in the stomach resulting from the rapid passage of contrast into the duodenum, despite the timing of MRCP immediately after pineapple juice administration. Our failure to achieve an improvement of pancreatic duct visualisation is likely because of the anatomical overlapping of the MPD by the stomach on conventional coronal maximal intensity projection reconstruction.^13,15^ There were no serious adverse events in this study, in keeping with prior studies that demonstrated that manganese from orally administered fruit juices are poorly absorbed and do not significantly elevate serum concentrations.^29^

Our failure to demonstrate significant suppression of gastric SI or improvement of pancreatic duct visualisation highlights the need for further refinement of our MRCP protocol, including optimisation of the dose and timing of pineapple juice administration. Nonetheless, our study provides a useful baseline for further work in this domain. It is expected that other centres in South Africa and Africa will be prompted to adopt a similar, affordable protocol. The ideal volume of pineapple juice has still to be defined and is likely dependent on the concentration of manganese in each preparation. Further work could thus involve spectrometric evaluation of manganese concentrations in locally available pineapple juice preparations. In clinical practice it may be useful to administer a minimum of 250 mL and up to the maximum tolerated volume of pineapple juice, given the relatively low cost of the product.

## Conclusion

This study demonstrated that a store-bought, conveniently packaged and easily consumable quantity of pineapple juice improves the quality of MRCP imaging, and specifically improves visualisation of the CBD.
